# Clinical Frailty Scale and 12-month adverse health outcomes among community-dwelling older adults in Vietnam: a prospective cohort study

**DOI:** 10.3389/fmed.2026.1910694

**Published:** 2026-09-03

**Authors:** Ha Bich Thi Trinh, Kien Gia To, Tan Van Nguyen

**Affiliations:** 1 Department of Geriatrics and Gerontology, University of Medicine and Pharmacy at Ho Chi Minh City, Ho Chi Minh City, Vietnam; 2 Comprehensive Geriatrics and Older Adults Leading Research Group, University of Medicine and Pharmacy at Ho Chi Minh City, Ho Chi Minh City, Vietnam; 3 Faculty of Public Health, University of Medicine and Pharmacy at Ho Chi Minh City, Ho Chi Minh City, Vietnam; 4 Department of Interventional Cardiology, Thong Nhat Hospital, Ho Chi Minh City, Vietnam

**Keywords:** Clinical Frailty Scale, community-dwelling, falls, frailty, hospitalization, mortality, older adults, Vietnam

## Abstract

**Background:**

Frailty is an important predictor of adverse health outcomes in older adults, but evidence from community settings in Vietnam remains limited. This study examined the association between frailty and adverse health outcomes among community-dwelling older adults.

**Methods:**

In this prospective cohort study, 1,208 community-dwelling older adults were assessed using the Clinical Frailty Scale (CFS) and followed for 12 months. The primary outcome was a composite of all-cause mortality, falls, and hospitalization. Multivariable logistic regression was used to evaluate the independent association between frailty and adverse outcomes.

**Results:**

Of the participants, 59.9% were robust, 7.8% vulnerable, 31.2% had mild-to-moderate frailty, and 1.2% had severe frailty. During follow-up, 23.6% experienced at least one adverse outcome. Event rates increased progressively across frailty categories, from 9.7% in robust participants to 64.3% in those with severe frailty (*P* for trend <0.001). After adjustment for demographic characteristics, lifestyle factors, multimorbidity, polypharmacy, and cognitive impairment, frailty remained independently associated with adverse outcomes (adjusted OR per one-point increase in CFS: 1.88, 95% CI: 1.67–2.11; *P* < 0.001).

**Conclusion:**

Frailty was highly prevalent and strongly associated with adverse health outcomes among community-dwelling older adults. These findings support the use of the Clinical Frailty Scale to identify community-dwelling older adults who may benefit from closer monitoring and targeted preventive care.

## Introduction

Population ageing is accelerating worldwide and has become a major public health challenge. According to the United Nations, the number of people aged 65 years and older is projected to more than double over the coming decades, with the most rapid increases occurring in low- and middle-income countries (1). Vietnam is experiencing a rapid demographic transition towards an ageing society, reflecting broader ageing trends observed across many low- and middle-income countries. In this context, simple approaches to identify older adults at risk of poor outcomes are increasingly needed in community health care.

Frailty is a multidimensional geriatric syndrome characterized by diminished physiological reserve and increased vulnerability to endogenous and exogenous stressors (2). Compared with robust older adults, frail individuals are more likely to experience falls, hospitalization, disability, institutionalization, and mortality (2–4). Frailty has increasingly been recognized as a clinical state distinct from chronological ageing and multimorbidity, providing important prognostic information beyond traditional risk factors (3). Consequently, frailty assessment has become an integral component of geriatric care and healthy ageing strategies worldwide (3, 4).

Several instruments have been developed to assess frailty, including the Frailty Phenotype and the Frailty Index (2, 3). Among these, the Clinical Frailty Scale (CFS), developed by Rockwood and colleagues, has gained widespread acceptance because of its simplicity, feasibility, and strong predictive performance (5). The CFS is a judgement-based frailty tool that incorporates comorbidity, function, cognition, and overall fitness into a single score ranging from 1 to 9 (5, 6). Because of its simplicity and feasibility, the CFS has been widely applied across clinical and community settings, and higher CFS scores have been associated with adverse health outcomes in older adults (6).

Despite growing recognition of frailty as a key determinant of healthy ageing, important knowledge gaps remain. Most studies evaluating the prognostic significance of frailty and the Clinical Frailty Scale have been conducted in hospitalized populations, long-term care facilities, or high-income countries (5, 6). Community-based longitudinal evidence remains comparatively limited, particularly in low- and middle-income countries (7).

In Vietnam, previous community-based studies have shown that frailty is highly prevalent among older adults and is associated with poorer health-related quality of life, multimorbidity, and other adverse health conditions (8, 9). However, these studies have primarily focused on estimating frailty prevalence and identifying associated factors rather than evaluating the longitudinal prognostic significance of frailty. Evidence examining whether frailty independently predicts subsequent adverse health outcomes among community-dwelling older adults in Vietnam remains limited. Such information is essential for community-based risk stratification and the development of targeted interventions to preserve health, function, and independence in later life.

Therefore, this prospective cohort study aimed to evaluate the association between frailty severity assessed using the Clinical Frailty Scale and 12-month adverse health outcomes among community-dwelling older adults in Vietnam.

## Methods

### Study design and setting

This prospective community-based cohort study was conducted among community-dwelling older adults residing in Binh Thanh District, Ho Chi Minh City, Vietnam. Baseline recruitment and assessment were conducted between November 2023 and April 2025, and participants were followed for 12 months after baseline assessment.

At baseline, trained investigators approached participants in person and conducted face-to-face interviews and standardized geriatric assessments in the community. Information on sociodemographic characteristics, lifestyle factors, medical history, comorbidities, medication use, functional status, and frailty was collected. Participants were subsequently followed for 12 months to ascertain adverse health outcomes.

The study was conducted and reported in accordance with the Strengthening the Reporting of Observational Studies in Epidemiology (STROBE) Statement for cohort studies (10).

### Participants

Community-dwelling adults aged 60 years or older residing in Binh Thanh District, Ho Chi Minh City, Vietnam, were eligible to participate.

A two-stage community-based sampling procedure was used. First, 30 of the 88 residential neighborhoods in Binh Thanh District were selected using probability proportional to size sampling, based on the number of older residents recorded by the Binh Thanh District Health Center. Second, lists of older residents in each selected neighborhood were obtained from the local Older People's Association or ward health station. A starting household was selected using computer-generated random numbers, after which potentially eligible older adults were approached in person by trained field investigators until the target sample for each neighborhood was reached.

Individuals were excluded if they were unable to provide informed consent and had no legally authorized representative available, had severe communication impairment precluding assessment, or had insufficient baseline or follow-up information for the study analyses.

Of 1,325 older adults assessed for eligibility, 62 declined participation, 30 did not complete the baseline assessment, and 25 had incomplete follow-up information. A total of 1,208 participants with complete baseline and 12-month follow-up data were included in the final analysis. All participants provided written informed consent before enrollment.

### Baseline assessment

At enrollment, all participants underwent a standardized multidimensional geriatric assessment conducted by trained healthcare personnel. Information on sociodemographic characteristics, including age, sex, educational attainment, marital status, and living arrangements, was collected. Lifestyle factors included smoking, alcohol consumption, and regular physical exercise. Medical history was characterized by the number of chronic diseases and the presence of multimorbidity, while medication use was assessed by the number of medications and the presence of polypharmacy. The number of falls during the 12 months preceding enrollment was also recorded, and previous falls were defined as at least one fall during this period. These variables were included in the descriptive analyses, and their univariable associations with the composite adverse health outcome were examined. A multidimensional geriatric assessment was performed. Functional status was evaluated using the Katz Activities of Daily Living (ADL) Index and the Lawton Instrumental Activities of Daily Living (IADL) Scale (11, 12). Cognitive function was assessed using the Mini-Mental State Examination (MMSE). Cognitive impairment was defined as an MMSE score <24 (13). Symptoms suggestive of sarcopenia were screened using the SARC-F questionnaire, with a score ≥4 indicating probable sarcopenia (14).

Multimorbidity was defined as the presence of two or more chronic medical conditions. Chronic conditions were assessed using a predefined list of physician-diagnosed diseases, including hypertension, diabetes mellitus, coronary artery disease, dyslipidemia, heart failure, atrial fibrillation, stroke, Parkinson's disease, chronic kidney disease, osteoarthritis, peptic disease, and chronic venous insufficiency. Polypharmacy was defined as the regular use of five or more medications (15, 16). These variables were collected because of their potential associations with frailty and adverse health outcomes in older adults.

### Frailty assessment

Frailty was assessed at baseline using the Clinical Frailty Scale (CFS), a validated 9-point global frailty instrument developed by Rockwood and colleagues (5). The CFS ranges from 1 (very fit) to 9 (terminally ill), with higher scores indicating greater frailty severity. Scores were assigned by trained assessors based on participants' usual health status, considering mobility, functional dependence, cognition, comorbidity burden, and overall fitness.

For descriptive analyses, participants were categorized according to CFS severity groups: robust (CFS: 1–3), vulnerable (CFS: 4), mild-to-moderate frailty (CFS: 5–6), and severe frailty (CFS: ≥7). In the primary analyses, the CFS was examined as an ordinal measure to evaluate whether increasing frailty severity was associated with a higher risk of 12-month adverse health outcomes. Categorical CFS groups were additionally used for descriptive and sensitivity analyses.

### Outcome assessment

Participants were followed prospectively for 12 months after enrollment, with outcome assessments conducted at 3, 6, 9, and 12 months. Outcome information was collected through structured telephone interviews with participants or cohabitating family members when participants were unable to provide information directly.

The primary outcome was a composite adverse health outcome, predefined as the occurrence of at least one of the following events during the 12-month follow-up period: all-cause mortality, falls, or hospitalization.

All-cause mortality was defined as death from any cause occurring during the study period. Falls were defined according to the World Health Organization definition as events resulting in a person coming to rest inadvertently on the ground, floor, or another lower level. Hospitalization was defined as any hospital admission occurring during follow-up.

Secondary outcomes comprised the individual components of the composite outcome, including all-cause mortality, falls, and hospitalization, which were analyzed separately.

### Statistical analysis

Continuous variables are presented as mean ± standard deviation (SD) or median with interquartile range (IQR), as appropriate according to data distribution. Categorical variables are presented as frequencies and percentages. Baseline characteristics were compared across CFS categories. Continuous variables were compared using one-way analysis of variance (ANOVA) or the Kruskal–Wallis test, whereas categorical variables were compared using the chi-square test or Fisher's exact test, as appropriate. The cumulative incidence of adverse health outcomes during the 12-month follow-up period was calculated according to frailty category. Because the primary outcome was defined as the occurrence of at least one adverse health outcome during the predefined 12-month follow-up period, rather than the time to the first event, univariable and multivariable logistic regression analyses were used to evaluate the association between frailty severity and adverse health outcomes. Associations are reported as odds ratios (ORs) with 95% confidence intervals (CIs). The primary exposure variable was frailty severity assessed using the CFS. Covariates included in multivariable models were selected *a priori* based on clinical relevance and evidence from previous literature.

Three multivariable models were constructed. Model 1 was adjusted for age and sex. Model 2 was additionally adjusted for educational attainment, physical activity, body mass index, multimorbidity, and polypharmacy. Model 3 further included cognitive impairment (MMSE <24). Multicollinearity among variables included in the fully adjusted model was assessed using tolerance values and variance inflation factors (VIFs). Functional measures (ADL and IADL), probable sarcopenia, and urinary incontinence were not included in the multivariable models because these variables are conceptually and clinically related to frailty and may represent components or consequences of the frailty construct, potentially leading to overadjustment.

Additional analyses were performed for individual outcome components, including all-cause mortality, falls, and hospitalization. Missing data were minimal (<5% for all study variables); therefore, complete-case analysis was performed without imputation. Statistical significance was defined as a two-sided *P* value <0.05. All analyses were performed using IBM SPSS Statistics version 27.0 (IBM Corp., Armonk, NY, USA).

### Ethical considerations

The study was conducted in accordance with the ethical principles of the Declaration of Helsinki. The study protocol was reviewed and approved by the Institutional Review Board of the University of Medicine and Pharmacy at Ho Chi Minh City, Vietnam (Approval No. 23720-ĐHYD; October 16, 2023). Written informed consent was obtained from all participants before enrollment. For participants with limited ability to provide information independently, consent and follow-up information were additionally obtained from a family member living with the participant when appropriate. All collected data were anonymized and treated confidentially throughout the study.

## Results

### Baseline characteristics

**Figure 1:**
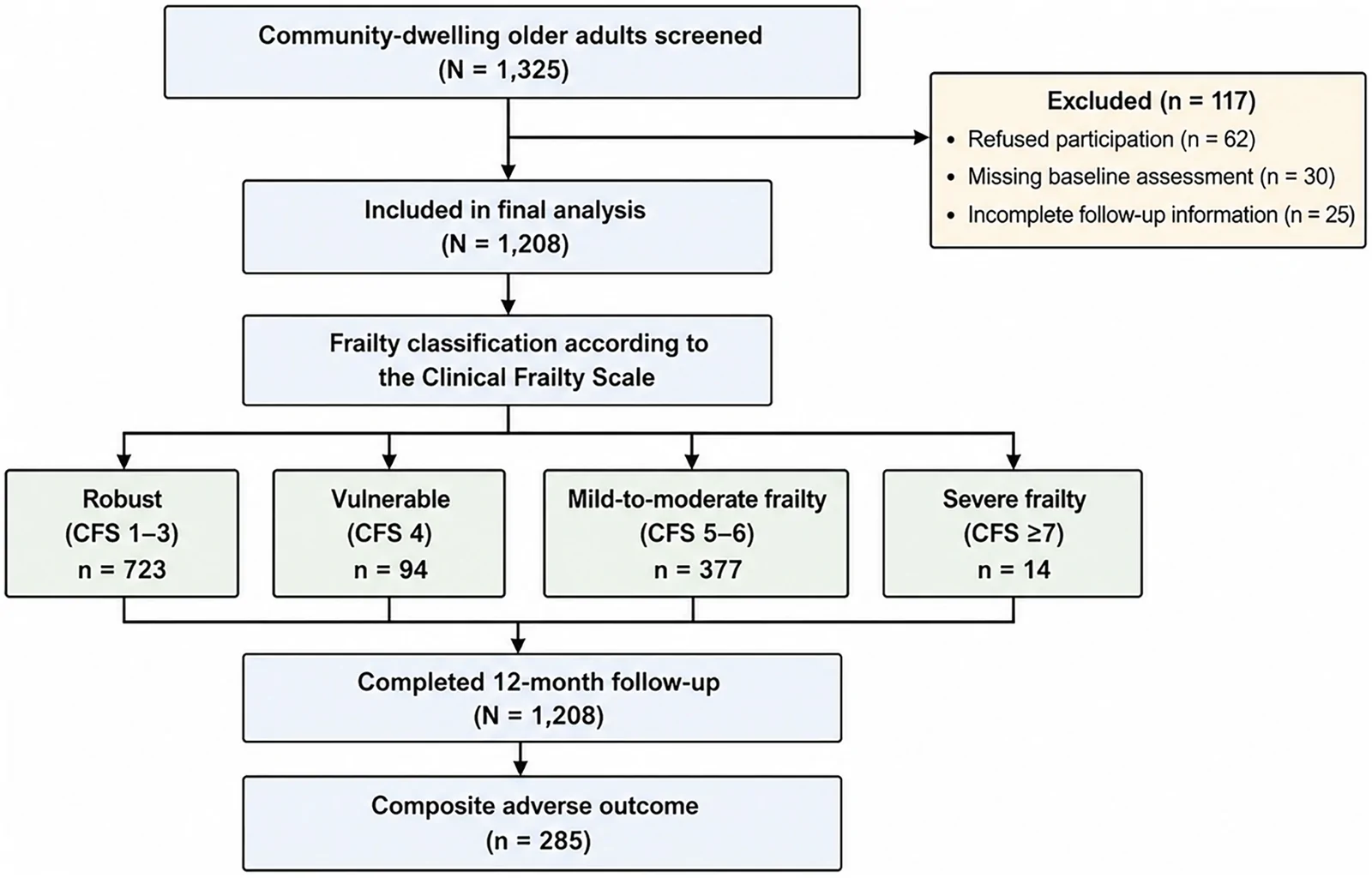
Study flow diagram.

The study flow and participant selection process are presented in Figure 1. Of 1,325 older adults initially assessed for eligibility, 1,208 participants were included in the final analysis after exclusion of individuals who declined participation, had missing baseline assessments, or had incomplete follow-up information. Participants were prospectively followed for 12 months to ascertain adverse health outcomes, including all-cause mortality, falls, and hospitalization. Baseline characteristics according to frailty severity categories are presented in Table 1.

**Table 1:** Baseline characteristics of participants according to frailty severity categories.

Characteristics	Overall (*N* = 1,208)	Robust (CFS 1–3) (*n* = 723)	Vulnerable (CFS 4) (*n* = 94)	Mild–moderate frailty (CFS 5–6) (*n* = 377)	Severe frailty (CFS ≥7) (*n* = 14)	*P* value
Demographic characteristics						
Age, years	71.7 ± 7.6	69.1 ± 5.5	71.9 ± 6.3	75.9 ± 8.4	88.2 ± 9.8	<0.001
Female sex, *n* (%)	814 (67.4)	463 (64.0)	70 (74.5)	271 (71.9)	10 (71.4)	0.024
Educational attainment, *n* (%)						<0.001
Primary school or less	280 (23.2)	111 (15.4)	31 (33.0)	131 (34.7)	7 (50.0)	
Secondary school	320 (26.5)	188 (26.0)	22 (23.4)	106 (28.1)	4 (28.6)	
High school	429 (35.5)	294 (40.7)	32 (34.0)	100 (26.5)	3 (21.4)	
College/university or higher	179 (14.8)	130 (18.0)	9 (9.6)	40 (10.6)	0 (0.0)	
Living alone, *n* (%)	69 (5.7)	48 (6.6)	3 (3.2)	18 (4.8)	0 (0.0)	0.293
Living with spouse, *n* (%)	637 (52.7)	422 (58.4)	49 (52.1)	162 (43.0)	4 (28.6)	<0.001
Lifestyle factors						
Current smoking, *n* (%)	137 (11.3)	95 (13.1)	9 (9.6)	32 (8.5)	1 (7.1)	0.116
Current alcohol consumption, *n* (%)	113 (9.4)	86 (11.9)	7 (7.4)	19 (5.0)	1 (7.1)	0.003
Regular physical exercise, *n* (%)	737 (61.0)	518 (71.6)	46 (48.9)	173 (45.9)	0 (0.0)	<0.001
Clinical characteristics						
Body mass index, kg/m^2^	22.9 ± 3.6	23.1 ± 3.3	23.5 ± 4.3	22.5 ± 4.0	21.3 ± 2.5	0.008
Number of chronic diseases	3 (1–4)	2 (1–4)	3 (2–5)	4 (2–5)	5 (2.2–5.8)	<0.001
Multimorbidity (≥2 diseases), *n* (%)	830 (68.7)	440 (60.9)	77 (81.9)	302 (80.1)	11 (78.6)	<0.001
Number of medications	4 (2–6)	4 (2–5)	5 (3–6)	5 (3–7)	6 (5–6)	<0.001
Polypharmacy (≥5 medications), *n* (%)	499 (41.3)	220 (30.4)	52 (55.3)	215 (57.0)	12 (85.7)	<0.001
Comprehensive geriatric assessment						
MMSE score	27 (25–29)	28 (27–29)	27 (25–28)	25 (23–28)	22 (14.5–24.8)	<0.001
Cognitive impairment (MMSE <24), *n* (%)	157 (13.0)	37 (5.1)	12 (12.8)	100 (26.5)	8 (57.1)	<0.001
ADL score	6 (6–6)	6 (6–6)	6 (6–6)	6 (5–6)	1.5 (0.2–5)	<0.001
ADL dependence, *n* (%)	188 (15.6)	0 (0.0)	2 (2.1)	173 (45.9)	13 (92.9)	<0.001
IADL score	8 (7–8)	8 (8–8)	8 (7–8)	6 (4–7)	1 (1–2.8)	<0.001
IADL impairment, *n* (%)	408 (33.8)	84 (11.6)	25 (26.6)	285 (75.6)	14 (100.0)	<0.001
SARC-F score	1 (0–3)	0 (0–1)	3 (2–4)	4 (1–6)	8 (7–8.8)	<0.001
Probable sarcopenia (SARC-F ≥ 4), *n* (%)	266 (22.0)	22 (3.0)	39 (41.5)	191 (50.7)	14 (100.0)	<0.001
Urinary incontinence, *n* (%)	183 (15.1)	3 (0.4)	4 (4.3)	166 (44.0)	10 (71.4)	<0.001
Falls in previous 12 months, *n* (%)	170 (14.1)	67 (9.3)	16 (17.0)	81 (21.5)	6 (42.9)	<0.001
Frailty						
Clinical Frailty Scale score	3 (2–5)	3 (2–3)	4 (4–4)	6 (5–6)	7 (7–7)	<0.001

ADL, activities of daily living; CFS, Clinical Frailty Scale; IADL, instrumental activities of daily living; MMSE, mini-mental state examination; SARC-F, Strength, Assistance with walking, Rise from a chair, Climb stairs, and Falls questionnaire.

Data are presented as mean ± standard deviation, median (interquartile range), or number (%), as appropriate. Percentages are calculated within each frailty category. *P* values were derived from one-way analysis of variance for normally distributed continuous variables, Kruskal–Wallis tests for ordinal or non-normally distributed continuous variables, and chi-square tests for categorical variables. Frailty categories were defined from the Clinical Frailty Scale as robust (CFS: 1–3), vulnerable (CFS: 4), mild–moderate frailty (CFS: 5–6), and severe frailty (CFS: ≥7).

Among the study population, 723 participants (59.9%) were classified as robust (CFS: 1–3), 94 (7.8%) as vulnerable (CFS: 4), 377 (31.2%) as having mild-to-moderate frailty (CFS 5–6), and 14 (1.2%) as having severe frailty (CFS: ≥7). Increasing frailty severity was associated with older age, lower educational attainment, lower levels of physical activity, lower body mass index, and higher prevalences of multimorbidity and polypharmacy. Participants with greater frailty severity also had lower MMSE, ADL, and IADL scores and higher prevalences of cognitive impairment, functional dependence, probable sarcopenia, urinary incontinence, and previous falls (Table 1).

### Incidence of adverse health outcomes

During the 12-month follow-up period, 285 participants (23.6%) experienced at least one adverse health outcome. Overall, 13 participants (1.1%) died, 160 (13.2%) experienced at least one fall, and 156 (12.9%) were hospitalized during follow-up (Table 2). The incidence of the composite adverse outcome increased markedly across frailty categories, from 9.7% among robust participants to 29.8% among vulnerable participants, 47.2% among those with mild-to-moderate frailty, and 64.3% among those with severe frailty (*P* < 0.001). Similar trends were observed for the individual outcome components. Overall, participants with greater frailty severity experienced higher rates of mortality, falls, and hospitalization during follow-up (Table 2).

**Table 2:** Twelve-month incidence of adverse health outcomes according to frailty severity categories.

Outcome	Overall (*N* = 1,208)	Robust (*n* = 723)	Vulnerable (*n* = 94)	Mild–moderate frailty (*n* = 377)	Severe frailty (*n* = 14)	*P* value
Composite adverse outcome, *n* (%)	285 (23.6)	70 (9.7)	28 (29.8)	178 (47.2)	9 (64.3)	<0.001
All-cause mortality, *n* (%)	13 (1.1)	1 (0.1)	0 (0.0)	7 (1.9)	5 (35.7)	<0.001
Falls, *n* (%)	160 (13.2)	33 (4.6)	18 (19.1)	106 (28.1)	3 (21.4)	<0.001
Hospitalization, *n* (%)	156 (12.9)	39 (5.4)	15 (16.0)	100 (26.5)	2 (14.3)	<0.001

CFS, Clinical Frailty Scale.

Data are presented as *n* (%).

*P* values were calculated using the chi-square test or Fisher's exact test, as appropriate.

Participants may have experienced more than one outcome during follow-up; therefore, the sum of individual outcome frequencies may exceed the number of participants with the composite outcome.

**Figure 2:**
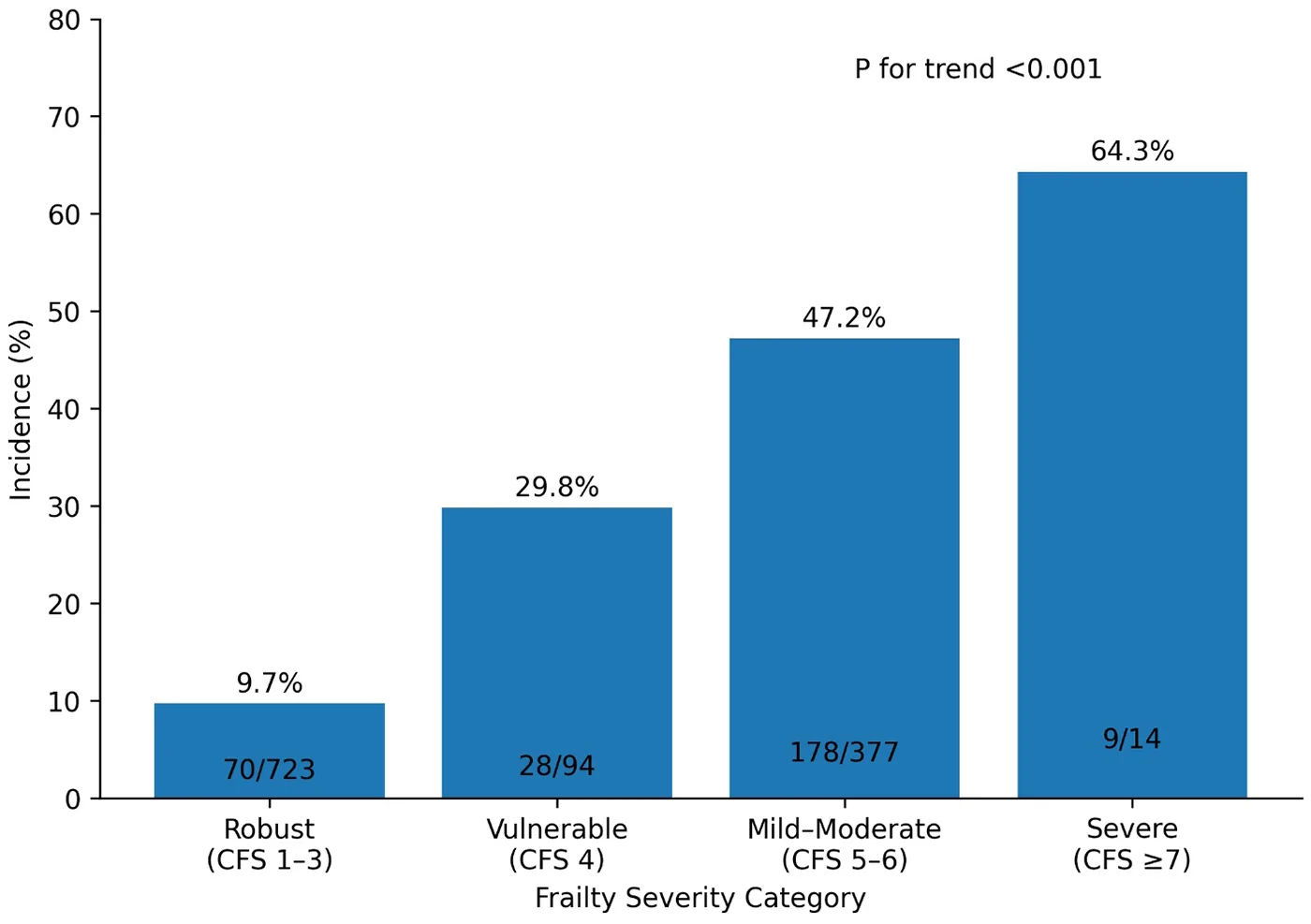
Twelve-month incidence of composite adverse health outcomes according to frailty severity categories.

Figure 2 illustrates the incidence of the composite adverse outcome according to frailty severity. The incidence of the composite adverse outcome increased progressively across frailty categories, from 9.7% among robust participants to 29.8% among vulnerable participants, 47.2% among those with mild-to-moderate frailty, and 64.3% among those with severe frailty (*P* for trend <0.001). The relationship demonstrated a clear dose–response pattern, with progressively higher event rates observed at increasing levels of frailty.

### Factors associated with composite adverse health outcomes

In univariable analyses, older age, lower educational attainment, physical inactivity, multimorbidity, polypharmacy, cognitive impairment, functional impairment, probable sarcopenia, urinary incontinence, previous falls, and higher frailty levels were significantly associated with composite adverse health outcomes during the 12-month follow-up period (Table 3).

**Table 3:** Factors associated with composite adverse health outcomes during 12-month follow-up: univariable analysis.

Variable	Odds ratio	95% CI	*P* value
Age (per year increase)	1.08	1.06–1.10	<0.001
Female sex	1.11	0.83–1.48	0.474
Educational attainment (per level increase)	0.76	0.68–0.86	<0.001
Living alone	1.25	0.72–2.15	0.428
Current smoking	0.66	0.42–1.05	0.077
Current alcohol consumption	0.55	0.32–0.93	0.026
No regular physical exercise	1.90	1.45–2.48	<0.001
Body mass index (per kg/m^2^ increase)	0.95	0.91–0.98	0.006
Multimorbidity (≥2 chronic diseases)	1.83	1.34–2.49	<0.001
Polypharmacy (≥5 medications)	2.25	1.72–2.94	<0.001
Cognitive impairment (MMSE <24)	3.23	2.28–4.57	<0.001
ADL dependence	5.74	4.13–7.98	<0.001
IADL impairment	3.55	2.70–4.68	<0.001
Probable sarcopenia (SARC-F ≥ 4)	4.33	3.23–5.82	<0.001
Urinary incontinence	4.74	3.41–6.60	<0.001
Falls during previous 12 months	3.40	2.43–4.77	<0.001
Clinical Frailty Scale (per one-point increase)	2.04	1.84–2.26	<0.001

ADL, activities of daily living; IADL, instrumental activities of daily living; MMSE, Mini-Mental State Examination; SARC-F, Strength, Assistance with walking, Rise from a chair, Climb stairs, and Falls questionnaire; CFS, Clinical Frailty Scale.

Data are presented as odds ratios with 95% confidence intervals. Univariable logistic regression was used to estimate the association between each baseline variable and the composite adverse health outcome.

The composite adverse health outcome was defined as the occurrence of all-cause mortality, falls, or hospitalization during the 12-month follow-up period.

Among these factors, ADL dependence (OR: 5.74, 95% CI: 4.13–7.98), urinary incontinence (OR: 4.74, 95% CI: 3.41–6.60), probable sarcopenia (OR: 4.33, 95% CI: 3.23–5.82), and higher Clinical Frailty Scale scores (OR: 2.04 per one-point increase, 95% CI: 1.84–2.26) showed the strongest associations with adverse outcomes.

### Multivariable analysis of factors associated with composite adverse health outcomes

Higher Clinical Frailty Scale scores remained independently associated with composite adverse health outcomes across all multivariable models (Table 4). In the fully adjusted model, each one-point increase in CFS was associated with an 88% higher odds of adverse outcomes during follow-up (aOR: 1.88, 95% CI: 1.67–2.11; *P* < 0.001). Frailty remained the strongest independent predictor of adverse health outcomes.

**Table 4:** Multivariable logistic regression analysis for composite adverse health outcomes during 12-month follow-up.

Variable	Model 1 aOR (95% CI)	*P* value	Model 2 aOR (95% CI)	*P* value	Model 3 aOR (95% CI)	*P* value
Age (per year increase)	1.03 (1.01–1.05)	0.010	1.02 (1.00–1.04)	0.045	1.02 (1.00–1.04)	0.074
Female sex	0.92 (0.66–1.26)	0.589	0.92 (0.66–1.28)	0.620	0.92 (0.66–1.28)	0.607
Educational attainment (per level increase)	—	—	0.96 (0.83–1.10)	0.553	0.97 (0.84–1.13)	0.727
No regular physical exercise	—	—	1.02 (0.75–1.39)	0.897	1.02 (0.74–1.39)	0.922
Body mass index (per kg/m^2^ increase)	—	—	0.96 (0.92–1.00)	0.071	0.96 (0.92–1.00)	0.067
Multimorbidity (≥2 chronic diseases)	—	—	0.96 (0.63–1.48)	0.869	0.97 (0.63–1.48)	0.873
Polypharmacy (≥5 medications)	—	—	1.35 (0.94–1.94)	0.105	1.35 (0.94–1.95)	0.102
Cognitive impairment (MMSE <24)	—	—	—	—	1.17 (0.81–1.71)	0.401
Clinical Frailty Scale (per one-point increase)	1.94 (1.74–2.17)	<0.001	1.89 (1.69–2.13)	<0.001	1.88 (1.67–2.11)	<0.001

MMSE, Mini-Mental State Examination; CFS, Clinical Frailty Scale.

Data are presented as adjusted odds ratios (aORs) with 95% confidence intervals (CIs).

Model 1 was adjusted for age, sex, and Clinical Frailty Scale.

Model 2 was adjusted for age, sex, educational attainment, regular physical exercise, body mass index, multimorbidity, polypharmacy, and Clinical Frailty Scale.

Model 3 was further adjusted for cognitive impairment (MMSE <24).

ADL dependence, IADL impairment, probable sarcopenia, and urinary incontinence were not included in the main multivariable models because these variables are conceptually and clinically related to frailty and may represent components or consequences of the frailty construct.

### Forest plot of independent predictors of adverse outcomes

**Figure 3:**
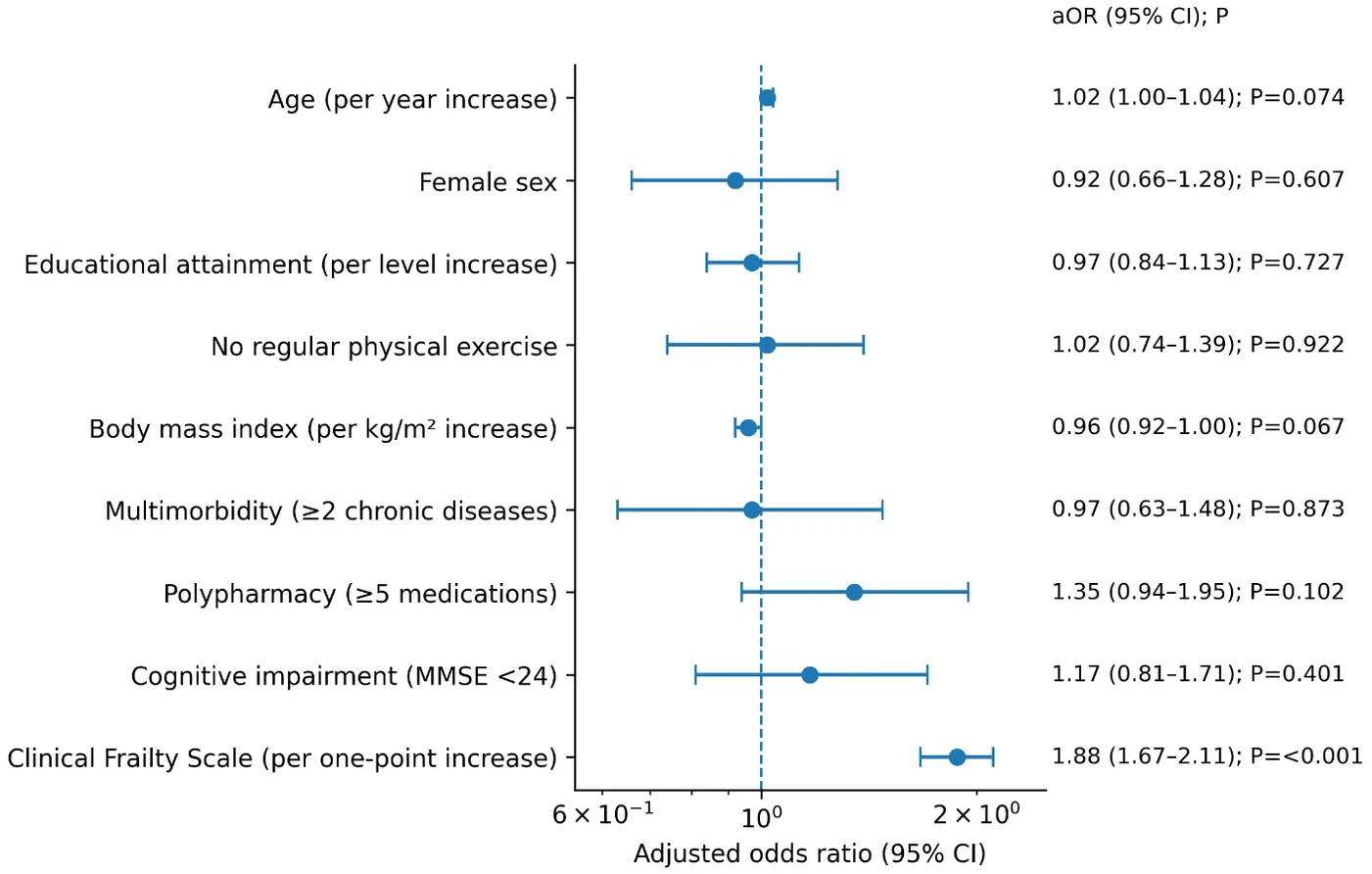
Forest plot of adjusted odds ratios for composite adverse health outcomes in the fully adjusted model. Adjusted odds ratios were derived from Model 3, which included age, sex, educational attainment, physical exercise, body mass index, multimorbidity, polypharmacy, cognitive impairment, and Clinical Frailty Scale score.

Figure 3 illustrates the adjusted associations between baseline characteristics and composite adverse health outcomes. Frailty remained the most robust independent predictor in the fully adjusted model, whereas the effects of most traditional demographic and clinical factors were attenuated after adjustment. The magnitude and consistency of the association between CFS and adverse outcomes support the prognostic importance of frailty assessment in community-dwelling older adults.

## Discussion

In this prospective community-based cohort study of 1,208 older adults followed for 12 months, frailty was highly prevalent and strongly associated with subsequent adverse health outcomes. Overall, 32.4% of participants were classified as frail, including 31.2% with mild-to-moderate frailty and 1.2% with severe frailty, while an additional 7.8% were categorized as vulnerable. During follow-up, 23.6% of participants experienced at least one adverse health outcome, including mortality, falls, or hospitalization. Importantly, the incidence of adverse outcomes increased progressively across frailty categories, from 9.7% among robust individuals to 29.8% among vulnerable participants, 47.2% among those with mild-to-moderate frailty, and 64.3% among those with severe frailty. Furthermore, each one-point increase in the Clinical Frailty Scale was independently associated with an 88% higher odds of adverse outcomes after adjustment for demographic characteristics, lifestyle factors, multimorbidity, polypharmacy, and cognitive impairment (adjusted OR: 1.88, 95% CI: 1.67–2.11). These findings highlight the burden of frailty in the community and suggest that the Clinical Frailty Scale may help identify older adults at higher risk of subsequent adverse events.

Frailty was highly prevalent in our cohort, affecting nearly one-third of participants, while an additional proportion of older adults were categorized as vulnerable. These findings indicate that approximately two out of every five community-dwelling older adults exhibited some degree of vulnerability or frailty. The prevalence observed in our study is broadly consistent with previous reports showing that frailty is a common condition among older adults living in the community, although substantial variation exists across populations, age distributions, socioeconomic contexts, and frailty assessment instruments. In a systematic review including 21 studies from high-income countries, Collard and colleagues reported a pooled frailty prevalence of 10.7% and a prefrailty prevalence of 41.6%, with considerable heterogeneity attributable to differences in age distribution, operational definitions, and study settings (17). Similar findings have also been reported in low- and middle-income countries, where a recent systematic review estimated substantially higher prevalence rates of frailty and prefrailty than those reported in many high-income settings, highlighting the influence of socioeconomic and healthcare-related factors on frailty burden (7). The higher prevalence observed in our cohort may partly reflect differences in demographic characteristics, socioeconomic conditions, and the use of the Clinical Frailty Scale, which captures multidimensional vulnerability and functional decline.

Participants with greater frailty severity in our study were older, less physically active, had lower educational attainment and body mass index, and exhibited a greater burden of multimorbidity and polypharmacy. Furthermore, frail individuals demonstrated poorer cognitive and functional performance, with lower MMSE, ADL, and IADL scores and higher prevalences of cognitive impairment, probable sarcopenia, urinary incontinence, and previous falls. These findings are consistent with the conceptual framework proposed by Fried et al. and Rockwood et al., in which frailty represents a state of increased vulnerability resulting from the accumulation of deficits across multiple physiological systems (18, 19). The observed clustering of multimorbidity, functional impairment, cognitive decline, and geriatric syndromes among frail participants further supports the multidimensional nature of frailty and underscores its close relationship with other geriatric conditions commonly encountered in community settings.

Our findings are consistent with a growing body of evidence demonstrating that frailty is a powerful predictor of adverse outcomes among older adults. Similar associations have been observed across different frailty instruments in large community-based cohorts, suggesting that the prognostic value of frailty remains robust regardless of the specific assessment tool used (20). In a meta-analysis of 35 cohort studies involving more than 1.2 million participants, Vermeiren et al. reported that frail older adults had a significantly higher risk of mortality, hospitalization, disability, and falls than their non-frail counterparts, with pooled relative risks ranging from approximately 1.5 to more than 2.0 depending on the outcome examined (21). Similarly, Kojima's meta-analysis showed that frailty was associated with a more than twofold increased risk of incident disability among community-dwelling older adults (pooled OR approximately 2.3), while prefrailty was also associated with a significantly elevated risk compared with robust individuals (22). These findings are in line with the marked gradient observed in our study, where the incidence of adverse outcomes increased from 9.7% among robust participants to 64.3% among those with severe frailty.

Our findings are also consistent with observations from the deficit accumulation model proposed by Rockwood and colleagues (19). In large population-based cohorts, higher frailty levels have been associated with progressively greater risks of hospitalization, institutionalization, and mortality (2). In the present study, each one-point increase in Clinical Frailty Scale score was associated with an 88% increase in the odds of experiencing an adverse outcome during follow-up, even after adjustment for age, multimorbidity, polypharmacy, cognitive impairment, and other potential confounders. This consistency across studies reinforces the clinical relevance of frailty assessment beyond conventional demographic and disease-based risk factors. Although frailty was strongly associated with adverse health outcomes, the observed all-cause mortality rate (1.1%) was relatively low compared with that reported in several previous cohorts of older adults. This finding should be interpreted in the context of our study population, which consisted of community-dwelling older adults with a mean age of 71.7 years, nearly 60% of whom were classified as robust according to the Clinical Frailty Scale. Therefore, the relatively low mortality likely reflects the characteristics of this relatively healthy community-based cohort rather than an absence of prognostic impact of frailty. Nevertheless, some degree of healthy participant selection cannot be entirely excluded and should be considered when interpreting the generalizability of the findings.

The mechanisms underlying the association between frailty and adverse outcomes are likely multifactorial. In our study, frail participants exhibited a higher burden of multimorbidity, polypharmacy, cognitive impairment, probable sarcopenia, functional dependence, urinary incontinence, and previous falls, suggesting that frailty reflects the cumulative effects of deficits across multiple physiological systems. According to the deficit accumulation model proposed by Rockwood and colleagues, the progressive accumulation of health deficits results in reduced physiological reserve and increased vulnerability to stressors, thereby predisposing older adults to falls, hospitalization, disability, and death (19). The persistence of frailty as an independent predictor after adjustment for multimorbidity, polypharmacy, and cognitive impairment further suggests that frailty captures dimensions of biological vulnerability that are not fully explained by individual diseases alone.

These findings have practical implications for community geriatric care in Vietnam. The Clinical Frailty Scale is simple, rapid, and feasible for use in primary care and community settings, requiring minimal resources while providing substantial prognostic information. Early identification of frail older adults may facilitate targeted interventions, including physical activity programs, nutritional support, medication review, fall prevention strategies, and comprehensive geriatric assessment. Given the rapid population ageing occurring in Vietnam and many other low- and middle-income countries, integrating frailty screening into community health programs may represent an important strategy for reducing adverse health outcomes and promoting healthy ageing.

### Strengths and limitations

This study has several strengths. First, the prospective cohort design enabled the temporal assessment of frailty and subsequent adverse health outcomes. Second, a relatively large sample of community-dwelling older adults was included, enhancing the robustness of the findings. Third, frailty was assessed using the Clinical Frailty Scale, a simple and validated instrument that is readily applicable in routine clinical practice. Finally, outcome data were systematically collected during the 12-month follow-up period, allowing comprehensive evaluation of clinically relevant adverse events.

Several limitations should also be acknowledged. First, participants were recruited from a single community setting, which may limit the generalizability of the findings to other populations. Second, frailty status was assessed only at baseline, and potential transitions between frailty states during follow-up were not evaluated. Third, although multiple confounders were adjusted for, residual confounding from unmeasured factors cannot be completely excluded. Finally, the use of a composite outcome may have masked differences in the associations between frailty and individual adverse events. Nevertheless, the consistency of the findings across adjusted models supports the robustness of the observed associations. Future studies are warranted to evaluate whether early identification and targeted management of frailty can reduce adverse health outcomes and support healthy ageing among community-dwelling older adults.

## Conclusion

In this prospective cohort of community-dwelling older adults, frailty was common and independently associated with adverse health outcomes during 12 months of follow-up. The risk of these outcomes increased progressively with frailty severity, and higher Clinical Frailty Scale scores remained independently associated with adverse health outcomes after adjustment for multiple demographic and clinical factors. These findings support the incorporation of frailty assessment into routine community-based geriatric care to facilitate early identification of older adults at increased risk and to inform targeted preventive interventions.

## Data Availability

The raw data supporting the conclusions of this article will be made available by the authors, without undue reservation.
