# Supplementary material for: Clinical Frailty Scale and 12-month adverse health outcomes among community-dwelling older adults in Vietnam: a prospective cohort study
Source: Front Med (Lausanne). 2026 Sep 3;13:1910694. doi: 10.3389/fmed.2026.1910694 (PMC13581966; doi:10.3389/fmed.2026.1910694)
Supplement: Supplementary file 1 [file Datasheet1.docx]

**STROBE Statement - Checklist**

Manuscript Title

Clinical Frailty Scale and 12-Month Adverse Health Outcomes Among Community-Dwelling Older Adults in Vietnam: A Prospective Cohort Study

| **Item** | **Recommendation** | **Location in Manuscript** |
| --- | --- | --- |
| 1a | Indicate the study design in the title or abstract | Title; Abstract |
| 1b | Provide an informative and balanced summary of the study and findings | Abstract |
| 2 | Explain the scientific background and rationale | Introduction |
| 3 | State specific objectives | Introduction |
| 4 | Present key elements of study design | Methods – Study Design and Setting |
| 5 | Describe the setting, locations, and relevant dates | Methods – Study Design and Setting |
| 6a | Describe eligibility criteria and participant selection methods | Methods – Participants |
| 6b | Give matching criteria, if applicable | Not applicable |
| 7 | Define outcomes, exposure variables, potential confounders, and effect modifiers | Methods – Frailty Assessment; Outcome Assessment |
| 8 | Describe data sources and methods of assessment | Methods – Baseline Assessment |
| 9 | Describe efforts to address potential sources of bias | Statistical Analysis; Discussion |
| 10 | Explain how study size was determined | Methods – Participants |
| 11 | Explain how quantitative variables were handled in the analyses | Statistical Analysis |
| 12a | Describe statistical methods, including methods used to control for confounding | Statistical Analysis |
| 12b | Describe methods used to examine subgroups and interactions | Not applicable |
| 12c | Explain how missing data were addressed | Statistical Analysis |
| 12d | Explain how loss to follow-up was addressed | Figure 1; Results |
| 12e | Describe sensitivity analyses, if performed | Not applicable |
| 13a | Report numbers of participants at each stage of the study | Figure 1; Results |
| 13b | Give reasons for non-participation at each stage | Figure 1 |
| 13c | Consider use of a flow diagram | Figure 1 |
| 14a | Describe characteristics of study participants | Table 1 |
| 14b | Indicate the number of participants with missing data for each variable of interest | Statistical Analysis |
| 14c | Summarize follow-up time | Methods; Results |
| 15 | Report outcome events during follow-up | Table 2 |
| 16a | Provide unadjusted and adjusted estimates with measures of precision | Tables 3 and 4 |
| 16b | Report category boundaries when continuous variables were categorized | Methods – Frailty Assessment |
| 16c | Consider translating estimates into absolute risk, if relevant | Table 2 |
| 17 | Report other analyses, if performed | Not applicable |
| 18 | Summarize key results with reference to study objectives | Discussion |
| 19 | Discuss limitations of the study | Discussion – Strengths and Limitations |
| 20 | Provide an overall interpretation of the results | Discussion |
| 21 | Discuss the generalizability of the findings | Discussion |
| 22 | Give the source of funding and the role of funders | Funding Statement |

The authors have completed this checklist in accordance with the STROBE Statement for reporting cohort studies.
